# Cross-Sectional and Longitudinal Associations between Body Mass Index and Cardiometabolic Risk Factors in Adolescents in a Country of the African Region

**DOI:** 10.1155/2013/801832

**Published:** 2013-08-22

**Authors:** Tanica Lyngdoh, Bharathi Viswanathan, Edwin van Wijngaarden, Gary J. Myers, Pascal Bovet

**Affiliations:** ^1^Institute of Social and Preventive Medicine, Lausanne University Hospital, CH-1010 Lausanne, Switzerland; ^2^Section of Noncommunicable Diseases, Ministry of Health, Victoria, Seychelles; ^3^Department of Community and Preventive Medicine, University of Rochester Medical Center, Rochester, NY, USA; ^4^Departments of Neurology, Pediatrics, and Environmental Medicine, University of Rochester Medical Center, Rochester, NY, USA

## Abstract

We assessed the association between several cardiometabolic risk factors (CRFs) (blood pressure, LDL-cholesterol, HDL-cholesterol, triglycerides, uric acid, and glucose) in 390 young adults aged 19-20 years in Seychelles (Indian Ocean, Africa) and body mass index (BMI) measured either at the same time (cross-sectional analysis) or at the age of 12–15 years (longitudinal analysis). BMI tracked markedly between age of 12–15 and age of 19-20. BMI was strongly associated with all considered CRFs in both cross-sectional and longitudinal analyses, with some exceptions. Comparing overweight participants with those having a BMI below the age-specific median, the odds ratios for high blood pressure were 5.4/4.7 (male/female) cross-sectionally and 2.5/3.9 longitudinally (*P* < 0.05). Significant associations were also found for most other CRFs, with some exceptions. In linear regression analysis including both BMI at age of 12–15 and BMI at age of 19-20, only BMI at age of 19-20 remained significantly associated with most CRFs. We conclude that CRFs are predicted strongly by either current or past BMI levels in adolescents and young adults in this population. The observation that only current BMI remained associated with CRFs when including past and current levels together suggests that weight control at a later age may be effective in reducing CRFs in overweight children irrespective of past weight status.

## 1. Introduction

Obesity is known to be associated with cardiovascular outcomes [1–3] and with elevated levels of several cardiometabolic risk factors (CRFs), including blood pressure and several metabolic factors [4]. The association between obesity and several CRFs has already been observed in children and adolescents both cross-sectionally [5–7] and longitudinally [8–10], but the data are limited. Furthermore, intervention studies through lifestyle interventions [11], and bariatric surgery [12] in children show that a reduction of body weight among obese children results in reduced levels of several CRFs, such as blood pressure [13–15], lipids [16–18], and insulin [19]. There are several methods to measure adiposity in children, including BMI, waist circumference, or the waist-to-hip ratio. There is continued debate on which indicator best predicts cardiovascular risk [20] but several studies suggest that body mass index (BMI) can adequately predict cardiovascular risk in adults, adolescents, and young adults [9, 21, 22].

There is, however, a relative scarcity of studies that have examined the relation between obesity and CRFs in population-based samples of children and adolescents (i.e., outside of the clinical setting). Furthermore, most such studies in children and adolescents have relied on a cross-sectional design, although a few did have a prospective design, but most such studies were conducted in high-income countries [8, 9]. Furthermore, only a few studies have simultaneously compared the relative strength of the associations between obesity and a broad set of CRFs including both physiological risk factors (e.g., blood pressure) and biological markers (e.g., blood lipids, glucose, and uric acid) in youths so that the associations between obesity and different CRFs can be directly compared.

In this study, we examined both the cross-sectional and longitudinal relationships between overweight and several CRFs in a population-based sample of adolescents and young adults. Data on CRFs were derived from a cohort study of children followed since their birth to examine the association between maternal fish consumption and the children's neurological development [23, 24]. In this cohort study, BMI and CRFs were measured for the first time on the seventh follow-up visit at the age of 19-20 years. We then linked the CRF data with BMI values measured at the age of 12–15 years previously obtained during a routine screening program in all schools of the country [25, 26]. The main objectives of the present study were to directly compare the associations between obesity and several CRFs, to compare the associations based on cross-sectional or longitudinal designs, and to examine the contribution of current and past weight statuses in the prediction of current CRF levels.

## 2. Methods 

### 2.1. Study Population

The Republic of Seychelles is a rapidly developing small island state in the Indian Ocean located east of Kenya. The population is mostly of African descent. Between February 1989 and February 1990, a cohort of 779 children was enrolled at the age of 6 months as part of the Seychelles Child Development Study (SCDS). Details of this cohort study have been previously described [24]. The SCDS was undertaken to assess the association between pre-natal exposure to methyl mercury from fish consumption and neurocognitive development in a sample of children representative of the general population. BMI and a broad set of cardiometabolic risk factors were measured for the first time in the seventh follow-up visit in 2008-2009 when the participants were aged 19-20 years (age range: 18.7–20.5; mean age: 19.5; SD: 0.5).

### 2.2. Linkage of Data

BMI at the age of 12–15 years was derived from a routine school-based surveillance program. BMI was measured when children were in the 7th and 10th grades and were aged approximately 12.7 years for children in the 7th grade (SD: 0.4; range: 11.6–13.4) and 13.9 years for children in the 10th grade (SD: 0.6; range: 12.1–15.9). The school screening program was conducted in all public and private schools in Seychelles under the auspices of the Ministries of Health and Education. The methods and results of this screening program have been published previously [25–27]. Linkage of BMI in the school-based screening program with CRFs measured at the age of 19-20 years within the SCDS study was based on the national identification number that is available for all Seychelles citizens. We had previously linked data from the SCDS with data from the school screening program in order to examine the relationship between exposure to prenatal mercury (SCDS) and blood pressure at the age of 12–15 years (school screening program) [28]. Informed consent was obtained from the subject and caregiver of every participant for both the SCDS and the school screening program. The school screening program and the SCDS were approved by the Research and Ethical Committee of the Ministry of Health, Victoria, Seychelles. In addition, the SCDS was approved by the Human Subjects Review Board at the University of Rochester, Rochester, NY, USA. 

### 2.3. Clinical Measurements

Measurement of anthropometrics at age of 12–15 years has been detailed previously [27]. Briefly, weight was measured without shoes and in light garments by trained school nurses in the schools, during regular school hours (8 am–2:30 pm), using electronic scales (Seca 870, Hamburg, Germany). Height was measured with a fixed stadiometer (Seca 208). The same weight scales and stadiometers were available in all schools and the instruments were regularly checked for accuracy by the screening nurses and further checks were preformed at least once per year by the manager of the screening program. BMI was calculated as weight (kg) divided by height (meters) squared. We used the average of the two values of BMI measured at ages 12 and 15 years, to reflect BMI in early adolescence. 

At the age of 19-20 years, all measurements took place between 8 a.m. and 10 a.m. at the SCDS study center. Weight was measured with a precision electronic scale (Seca 870) and height was measured with a fixed stadiometer (Seca 208). The instruments were regularly checked for accuracy. BP was measured using a validated oscillometric automated device (Omron M5). Three readings were measured by a nurse, on the left arm, and using an appropriately sized cuff. Readings were taken at intervals of at least 1 minute between the BP readings after the participant had been sitting for at least 5 minutes. Systolic BP (SBP) and diastolic BP (DBP) were based on the average of the three readings. 

### 2.4. Biological Samples

The methods used to measure venous blood taken at the age of 19-20 years have been described previously [21]. Briefly, venous blood samples were collected after an overnight fast. Glucose, total cholesterol, HDL-cholesterol, triglycerides, and uric acid were measured at the central laboratory of the main hospital in Seychelles (Seychelles Hospital), using standard enzymatic methods (Konelab T series reagents, Thermo Scientific) with a Thermo Konelab 30 automatic analyzer (Konelab Corp., Espoo, Finland). LDL-cholesterol was calculated using the Friedewald formula.

### 2.5. Statistical Analyses

Among the 423 adolescents who had complete data on CRFs and BMI at the age of 19-20 years in the SDCS (77% of 549 children seen at the age of 19-20 years), 390 (92%) also had data on BMI at the age of 12–15 years; hence, this study includes 390 participants. Analyses were conducted separately in males and females because of substantial sex differences in the distribution of several variables, including BMI and several CRFs, and the possibility that the relations between BMI and CRFs may differ according to sex. In order to examine the associations between BMI and CRFs, we considered three categories of body weight: (1) “low” BMI was defined as a BMI below the median values for age and sex (both at age of 19-20 years and at age of 12–15 years); (2) “elevated” BMI was defined as BMI above the age- and sex-specific median values of BMI and below the age- and sex-specific BMI cut-off values for “overweight”; and (3) “overweight.” Overweight at age of 12–15 years was defined according to the age- and sex-specific IOTF criteria of overweight [29]. Overweight in persons aged 18 years and above follows guidelines in adults and are defined as a BMI of 25 or above [30]. In both adolescents and young adults our cut-off values for “overweight” encompass both “overweight” and “obesity” categories. We did not distinguish between “overweight” and “obesity” because of an insufficient number of participants. We also conducted analyses examining the relationship between CRFs and joint categories of body weight at both age of 12–15 years and at age of 19-20 years (e.g., high-high, low-low, high-low, low-high) but the sample sizes in some categories (high-low, and low-high) were too small for meaningful statistical inference (results are available from the authors). We tabulated the mean values and 95% confidence intervals of CRFs according to these three categories of BMI separately in males and females. In order to directly compare the magnitude of the differences of the mean values of CRFs between these three BMI categories, we plotted the percent changes between values in the “elevated weight” and “overweight” categories compared to participants with low body weight. 

We also provide results in terms of odds ratios of elevated levels of CRFs according to the three defined categories of BMI, at age of 12–15 years or at age of 19-20 years, as determined by logistic regression. Regression models were not adjusted for other covariates, since the aim of the study was to examine the performance of BMI to predict CRFs. We defined dichotomized categories of CRFs in youths aged 19-20 years based on recent guidelines for subjects aged 18–21 years [31]. However, because only 4% of subjects had triglycerides ≥1.3 mmol/L, we used a lower cutoff (≥1.0 mmol/L) for this parameter which resulted in a prevalence of 12%. There is no widely used cutoff for serum uric acid in adolescents or young adults so we defined elevated levels as levels above the sex-specific 80th percentile. The cut-off values for the dichotomized categories of CRFs appear in Table 3.

We also examined the linear relationship between CRFs and BMI (used as a continuous variable) for both the crosssectional (BMI and CRFs measured at the age of 19-20 years) and longitudinal (BMI measured at the age of 12–15 years and CRFs measured at the age of 19-20 years) associations using linear regression, separately in males and females. Analyses were done separately for males and females. We also examined regression analysis models that included both BMI at the age of 12–15 years and BMI at the age of 19-20 years to determine how well the BMI predicted the defined outcomes at each age (i.e., to examine the association between CRFs and BMI at age of 19-20 years given a certain BMI at age of 12–15 years and, inversely, the association between CRFs and BMI at age of 12–15 years given a certain BMI at age of 19-20 years). To permit a direct comparison of the results based on cross-sectional and longitudinal analyses, we used standardized regression coefficients, which express change in the considered outcomes associated with a 1 standard deviation change in the exposure (BMI). Analyses were performed using Stata 11.2. All analyses were done among the 390 children who had complete data at age of 12–15 years and at age of 19-20 years. We used two-sided tests and considered statistical significance as *P* < 0.05.

## 3. Results

Table 1 presents the mean values and standard deviations of BMI and CRFs as well as the prevalence of the defined categories of BMI in participants aged 12–15 years and aged 19-20 years, separately in males and females. The proportion of participants who were overweight in the school evaluations was 18.9% among males and 19.5% among females aged 12–15 years. At age of 19-20 years, 12.6% of males and 27.9% of females were overweight (this also includes obesity). BMI and several CRFs differed substantially between males and females at both the age of 12–15 years and at the age of 19-20 years. The Spearman correlation coefficients between BMI at age of 12–15 years and BMI at age 19-20 years were 0.69 (*P* < 0.001) in males and 0.83 (*P* < 0.001) in females, which suggests a high degree of tracking of BMI over age.

The distribution of the CRFs at the age of 19-20 years according to our three defined BMI categories at the age of 12–15 years and at the age of 19-20 years is presented in Table 2. A significant positive linear trend was found between categories of BMI and most CRFs at both ages. Associations did not reach statistical significance for triglycerides and glucose in males and LDL-cholesterol, HDL-cholesterol and triglycerides in females at age of 12–15 years and glucose in males at age 19-20 years.

**Figure 1:**
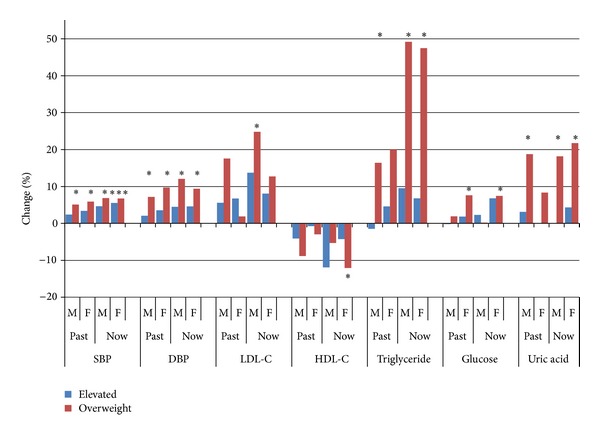
Relative difference (in percent) in the mean levels of cardiometabolic risk factors at the age of 19-20 years according to BMI categories measured at the age of 12–15 years (past) or at the age of 19-20 years (now). The reference BMI category (“low”) includes children whose BMI is below the age- and sex-specific median; “overweight” (which also includes obesity) is defined according to standard guidelines and “elevated” BMI refers to BMI values between the “low” and “overweight” categories. SBP: systolic blood pressure; DBP: diastolic blood pressure; LDL-C: LDL cholesterol; HDL-C: HDL cholesterol; M: male; F: female; past: BMI measured at the age of 12–15 years; now: BMI measured at age of 19-20 years. The stars indicate association with statistical significance of *P* < 0.05.

Figure 1 shows the percent changes in mean CRFs levels in males and females comparing the categories “elevated BMI” and “overweight” with “BMI below the median.” Overall, these changes were quite similar whether based on the longitudinal or cross-sectional analyses. However, the cross-sectional associations tended to be slightly larger than the longitudinal ones. The magnitude of the percent changes tended to be larger for several metabolic markers than for BP. However, the associations for the metabolic CRFs were not always statistically significant, which is consistent with larger variability (e.g., larger SD) for metabolic CRFs than for BP (Table 1).

Table 3 shows the odds ratios (ORs) for the presence of elevated CRFs in participants with elevated BMI and with overweight compared to participants with low BMI. Comparing participants with overweight versus participants with low BMI, these ORs were as high as 4.0 (95% CI: 1.5–10.4) for LDL-cholesterol among males aged 12–15 years and 8.0 (95% CI: 2.3–28.6) for triglycerides among males aged 19-20 years. Similar to the results from the stratified (Table 2) and linear regression (Table 3) analyses, the magnitude of the associations based on dichotomous categories of CRFs (i.e., ORs for the presence of elevated CRFs versus non elevated CRFs) in relation to BMI categories was generally not different whether the results were derived from longitudinal analysis (BMI measured at the age of 12–15 years and CRFs measured at the age of 19-20 years) or cross-sectional analysis (BMI and CRFs both measured at the age of 19-20 years), with some exceptions.

Table 4 shows the standardized linear coefficients derived from linear regression between CRFs measured at the age of 19-20 years and BMI measured either at the age of 12–15 years or at the age of 19-20 years, in males and females separately. Most of the associations were highly significant for most CRFs at both the earlier and later ages. This is in line with what was found for stratified analysis (Table 2, Figure 1). The associations tended to be slightly stronger for the cross-sectional associations between BMI and CRFs measured at 19-20 years than for the longitudinal associations (BMI measured at the age of 12–15 years and CRFs measured at the age of 19-20 years). Consistent with results of stratified analysis (Table 2), no associations were found for glucose in males (with BMI measured at any age) as well as for LDL-cholesterol and HDL-cholesterol in females when BMI was measured at the age of 12–15 years.

In analyses including BMI measured at both ages, only BMI measured at the age of 19-20 years was significantly associated with CRFs, but not BMI measured at the age of 12–15 years. The results may be sensitive to the variability in the measurements of the exposure variable and reflect that BMI was measured more accurately during the research evaluation at 19-20 years (highly standardized protocol with few observers and scales). However, the standard deviations of BMI were not substantially different whether BMI was measured when participants were aged 12–15 years or 19-20 years. These results suggest that current BMI matters more than past BMI when predicting current levels of CRFs.

## 4. Discussion

We found that elevated levels of BP and several metabolic risk factors measured at the age of 19-20 years are predicted by either current BMI or BMI levels measured several years earlier (age of 12–15 years). Furthermore, when relating CRFs at the age of 19-20 years toboth current and past BMI levels, only current levels were significantly associated with CRFs. This suggests that a life course approach to weight control is needed but that current weight status is particularly important in order to limit impact of adiposity on CRFs. This is the first study to examine both the cross-sectional and longitudinal associations between obesity and a broad panel of CRFs in a population-based sample of adolescents and young adults in the African region.

Our findings of strong associations between obesity and several CRFs in adolescents and young adults are consistent with earlier studies and holds true whether analyses are cross-sectional [5–7] or longitudinal [8–10]. The CRFs most consistently associated with obesity in youth include high blood pressure, dyslipidemia, hyperinsulinemia, and insulin resistance [9, 10, 32]. What our study adds, is that a large impact of obesity on several CRFs can be observed both cross-sectionally and longitudinally in adolescents and young adults in a country in the Africa region. These data provide further useful evidence for the need to address obesity and its impact on CRFs in youth in the African region.

The increase in mean levels of CRFs in a dose-response manner across categories of BMI is consistent with previous studies showing a strong association and clustering of obesity with CRFs in young people. It is interesting to note, from our stratified analysis (Table 2), that the relation between BMI and mean levels CRFs seemed to be linear (i.e., an effect was also seen with the intermediate “elevated” category) in the case of BP, while the impact of BMI on several metabolic CRFs was most apparent only in the highest BMI category (i.e., overweight, which also includes obesity). An implication of these findings is that interventions targeting the entire population are useful for blood pressure control at the population level while abnormal levels of biological CRFs might be better addressed by targeting obese adolescents and young adults.

The question of whether BMI or different obesity indicators would perform differently in their ability to predict CRFs is still ongoing. Based on data of participants aged 19-20 years (SCDS), we previously showed that BMI was at least as adequate as several other adiposity markers to predict mean levels of CRFs [21]. We found in this study that the magnitude of the associations between overweight and CRFs was important, with odds ratios of elevated levels of CRFs associated with overweight being larger than 2-3 for most CRFs. These results are consistent with findings in other studies among young people, for example, the Bogalusa Heart Study in the USA, in which a BMI larger than the 95th percentile was associated with odds ratios of 2.4, 4.5, 3.0, 7.1, and 12.1 for raised diastolic BP, systolic BP, LDL-cholesterol, triglycerides, and insulin concentration, respectively, and 3.4 for low HDL cholesterol [8]. Overweight was also related strongly to lipid markers among children aged 6–11 years in Qatar [7]. Of note, we found strong associations between overweight in adolescents and young adults and virtually all components of the metabolic syndrome. The magnitude of the observed associations and the broad scope in terms of the many factors involved stress the importance of weight control programs in youth.

It is interesting to note that the association between BMI and CRFs was almost as strong in our study when BMI was measured 4–6 years before measuring the CRF outcomes (longitudinal design) as when they were measured at the same time (cross-sectional design). Analyses based on the longitudinal design strengthen the possibility that elevated body weight is causally associated with elevated levels of CRFs. Confounding or reverse causation is unlikely to account for these findings (e.g., high BP or dyslipidemia is unlikely to be the cause of obesity). It is remarkable, however, that the longitudinal associations between BMI and CRFs were quite strong in comparison to the cross-sectional associations. This reinforces the conclusion that overweight is very likely an important issue for cardiovascular health and that weight control needs be addressed early in life.

The finding that only BMI at age of 19-20 years remained a strong predictor of CRFs in a regression model including both BMI at age of 12–15 years and BMI at age of 19-20 years suggests that current BMI is particularly important in relation to the prediction of blood pressure and metabolic risk factors. Conclusions must, however, be carefully drawn on these results in view of possible methodological issues, for example, the possibility that that BMI was measured with less precision at age of 12–15 years in the context of a routine screening than at age of 19-20 years in a study with highly standardized methods. Nonetheless, this finding (i.e. that CRFs are strongly associated with current BMI but not with BMI measured several years earlier when considering both weight statuses together) implies the important role of current versus past BMI in relation to current CRFs levels. This is welcome news since current weight, not past weight, can possibly be addressed through interventions. However, overweight tends to track over age, and children who were overweight when they were younger are largely the same children who are overweight when they are older. This is confirmed by the Spearman correlation coefficient of BMI at age of 12–15 years and at age of 19-20 years. The larger importance of current versus past weight has been emphasized in previous research. Several longitudinal studies of both past and current weights have shown that obesity in childhood has only limited impact when assessing cardiovascular health in adulthood [33, 34], but other studies have found an impact of obesity in young people on both risk factors [9, 10] and actual CVD outcomes [35–37] in adulthood. Overall, our findings that CRFs are associated with current and past BMI, and that BMI tracks over time, support a life course approach to obesity including both population-based interventions aimed at preventing the occurrence of overweight in the population at all ages and individual-based interventions targeting overweight among adolescents and young adults.

The distribution of several CRFs was markedly different between sexes. The observation of higher uric acid blood levels in males compared to females was expected [38] and consistent with the uricosuric effect of estrogens in females, which confers a protective effect on several metabolic CRFs [39]. A higher SBP in males compared to females may be related to males being generally taller since height is an important determinant of BP in adolescents and young adults [40]. We found a significant association between BMI and glucose only in females even though males had higher blood glucose levels than females. A possible explanation for the observed sex differences could be the greater adiposity, in particular fat mass, in females than in males [41]. The influence of abdominal fat, which is particularly active metabolically [42], may not be fully captured by the measurement of BMI [43] and might be a key determinant of some of the observed sex differences.

Our study has several strengths, including the population-based sample; a fairly large sample size for a study including blood markers among healthy adolescents and young adults; a broad panel of CRF markers considering the study included healthy participants; analyses based on both longitudinal and cross-sectional designs; and the presence of a minimal number of potential confounders, comorbid conditions, and related treatments at this early age. There are also some potential limitations. Accuracy of BMI measurements at the age of 12–15 years may be less than optimal in the context of routine school screening programs. In addition, although participants were asked to fast, they may not have been all fasting when blood was collected at the age of 19-20 years. A larger sample size would also have been useful to assess the associations separately among overweight and obese children and in order to generate different cohorts with sufficient numbers of children who either gained or lost weight between age of 12–15 and age of 19-20 and the subsequent effects on CRFs at the age of 19-20 years.

In conclusion, we found strong associations between adolescents and young adults who were overweight and several CRFs in both cross-sectional and longitudinal analyses. These findings indicate that a life course approach to weight control with interventions as early as possible is warranted but that weight control even at a later age is equally or perhaps more efficacious in reducing cardiovascular risk. These findings in a country of the African region extend previous similar findings in high-income countries and highlight the adverse consequences of obesity on CRFs in adolescents and young adults in all regions.

## Figures and Tables

**Table 1:** Characteristics of the participants at the age of 12–15 years and at the age of 19-20 years.

	Males (*n* = 175)	Females (*n* = 215)	*P* value
*Age 12–15 years *			
Mean age	13.88 (0.62)	13.89 (0.53)	
Age range	12.01–15.98	12.01–14.57	
BMI (kg/m^2^)	19.35 (3.31)	20.29 (3.95)	0.013
*Categories of BMI *			
Overweight	33 (18.86)	42 (19.53)	
Elevated weight	43 (24.57)	77 (35.81)	
Low weight	99 (56.57)	96 (44.65)	0.035
*Age 19 years *			
Mean age	19.51 (0.33)	19.51 (0.29)	
Age range	18.8–20.4	18.8–20.5	
BMI (kg/m^2^)	21.57 (3.47)	22.71 (5.45)	0.017
*Categories of BMI *			
Overweight	22 (12.57)	60 (27.91)	
Elevated weight	62 (35.43)	51 (23.72)	
Low weight	91 (52.00)	104 (48.37)	<0.001
*Cardiovascular risk factors *			
SBP (mmHg)	121.23 (10.74)	110.49 (9.75)	<0.001
DBP (mmHg)	67.36 (7.80)	69.06 (7.90)	0.034
LDL-cholesterol (mmol/L)	2.44 (0.77)	2.74 (0.84)	<0.001
HDL-cholesterol (mmol/L)	1.43 (0.41)	1.35 (0.39)	0.038
Triglycerides (mmol/L)	0.69 (0.28)	0.69 (0.35)	0.963
Glucose (mmol/L)	5.22 (0.58)	4.96 (0.54)	<0.001
Uric acid (mmol/L)	0.35 (0.08)	0.24 (0.07)	<0.001

BMI: body mass index; SBP: systolic blood pressure; DBP: diastolic blood pressure; LDL-cholesterol: low-density lipoprotein cholesterol; HDL-cholesterol: high-density lipoprotein.

Results are presented as mean (standard deviation) or as number (percentage).

**Table 2:** Distribution of mean levels (and 95% confidence intervals) of cardiometabolic risk factors at the age of 19 years according to categories of body mass index measured at the age of 12–15 years or at the age of 19-20 years.

		Age 12–15 years			Age 19-20 years		
		Overall	M	F	Overall	M	F
SBP (mmHg)	Low weight	113.7 (112.2–115.4)	119.4 (117.3–121.5)	107.9 (106.1–109.8)	112.3 (110.7–113.9)	118.3 (116.0–120.5)	107.01 (105.3–108.8)
	Elevated weight	115.4 (113.4–117.4)	122.2 (119.1–125.4)	111.6 (109.5–113.7)	118.9 (116.9–120.9)	123.8 (121.3–126.2)	113.0 (110.48–115.6)
	Overweight	119.2 (116.4–122.0)	125.5 (121.5–129.5)	114.3 (111.1–117.5)	117.5 (115.2–119.9)	126.4 (121.9–130.8)	114.3 (111.9–116.7)
*P*-trend		*0.002 *	*0.015 *	*<0.001 *	*<0.001 *	*<0.001 *	*<0.001 *
DBP (mmHg)	Low weight	66.5 (65.5–67.6)	66.1 (64.6–67.7)	66.9 (65.5–68.4)	66.0 (65.0–67.1)	65.3 (63.8–66.9)	66.6 (65.1–68.1)
	Elevated weight	68.7 (67.3–70.0)	67.5 (65.3–69.8)	69.3 (67.6–71.0)	68.9 (67.5–70.3)	68.3 (66.3–70.3)	69.6 (67.6–71.7)
	Overweight	72.3 (70.5–74.1)	70.9 (68.2–73.5)	73.4 (70.9–76.0)	72.9 (71.3–74.6)	73.2 (70.2–76.2)	72.8 (70.9–74.8)
*P*-trend		<0.001	0.003	<0.001	<0.001	<0.001	<0.001
LDL-cholesterol (mmol/L)	Low weight	2.50 (2.39–2.61)	2.33 (2.20–2.46)	2.67 (2.51–2.84)	2.44 (2.34–2.55)	2.26 (2.12–2.39)	2.60 (2.46–2.75)
	Elevated weight	2.71 (2.54–2.87)	2.46 (2.20–2.71)	2.85 (2.64–3.05)	2.68 (2.51–2.84)	2.57 (2.36–2.78)	2.81 (2.54–3.07)
	Overweight	2.73 (2.54–2.92)	2.74 (2.42–3.07)	2.72 (2.48–2.96)	2.90 (2.71–3.09)	2.82 (2.43–3.20)	2.93 (2.71–3.16)
*P*-trend		*0.009 *	*0.010 *	*0.428 *	*<0.001 *	*0.001 *	*0.020 *
HDL-cholesterol (mmol/L)	Low weight	1.42 (1.36–1.48)	1.47 (1.40–1.55)	1.36 (1.27–1.45)	1.46 (1.40–1.51)	1.51 (1.42–1.59)	1.41 (1.33–1.49)
	Elevated weight	1.37 (1.30–1.44)	1.41 (1.28–1.55)	1.35 (1.26–1.44)	1.34 (1.26–1.42)	1.33 (1.23–1.43)	1.35 (1.23–1.47)
	Overweight	1.33 (1.25–1.42)	1.34 (1.19–1.50)	1.32 (1.22–1.42)	1.29 (1.21–1.37)	1.43 (1.24–1.63)	1.24 (1.15–1.32)
*P*-trend		*0.062 *	*0.051 *	*0.596 *	*0.001 *	*0.058 *	*0.008 *
Triglycerides (mmol/L)	Low weight	0.66 (0.62–0.70)	0.67 (0.62–0.72)	0.65 (0.60–0.71)	0.63 (0.60–0.66)	0.63 (0.58–0.67)	0.63 (0.58–0.67)
	Elevated weight	0.67 (0.61–0.73)	0.66 (0.59–0.73)	0.68 (0.59–0.77)	0.64 (0.60–0.69)	0.69 (0.63–0.74)	0.59 (0.53–0.66)
	Overweight	0.78 (0.68–0.88)	0.78 (0.63–0.93)	0.78 (0.65–0.92)	0.89 (0.78–1.00)	0.94 (0.72–1.15)	0.87 (0.74–1.01)
*P*-trend		*0.165 *	*0.405 *	*0.211 *	*<0.001 *	*<0.001 *	*0.007 *
Fasting glucose (mmol/L)	Low weight	5.03 (4.96–5.11)	5.21 (5.09–5.33)	4.86 (4.77–4.95)	5.00 (4.92–5.08)	5.18 (5.05–5.32)	4.84 (4.76–4.93)
	Elevated weight	5.04 (4.94–5.13)	5.20 (5.01–5.39)	4.95 (4.84–5.05)	5.13 (5.03–5.22)	5.30 (5.17–5.43)	4.92 (4.81–5.04)
	Overweight	5.26 (5.11–5.42)	5.31 (5.12–5.50)	5.23 (4.99–5.47)	5.20 (5.05–5.34)	5.19 (4.93–5.43)	5.20 (5.02–5.38)
*P*-trend		*0.034 *	*0.569 *	*0.004 *	*0.008 *	*0.509 *	*<0.001 *
Uric acid (mmol/L)	Low weight	0.28 (0.27–0.29)	0.32 (0.31–0.34)	0.24 (0.23–0.26)	0.27 (0.26–0.29)	0.33 (0.31–0.34)	0.23 (0.22–0.24)
	Elevated weight	0.27 (0.26–0.29)	0.33 (0.31–0.35)	0.24 (0.22–0.25)	0.29 (0.27–0.30)	0.33 (0.32–0.35)	0.24 (0.22–0.25)
	Overweight	0.31 (0.29–0.34)	0.38 (0.35–0.41)	0.26 (0.24–0.29)	0.31 (0.29–0.33)	0.39 (0.34–0.44)	0.28 (0.26–0.29)
P-trend		*0.080 *	*0.002 *	*0.078 *	*0.011 *	*0.008 *	*<0.001 *

SBP: systolic blood pressure; DBP: diastolic blood pressure; LDL-cholesterol: low-density lipoprotein cholesterol; HDL-cholesterol: high-density lipoprotein cholesterol.

Elevated weight is defined for weight above sex- and age-specific medians and below cut off for overweight.

**Table 3:** Odds ratios for the association between cardiometabolic risk factors at the age of 19-20 years and body mass index categories measured at the age of 12–15 years or at the age of 19 years.

	12–15 years		19-20 years	
	Males (n = 175)	Females (n = 215)	Males (n = 175)	Females (n = 215)
Blood pressure ≥120/80 mmHg (M: 53.1%; F: 21.9%)				
Low BMI	1	1	1	1
Elevated BMI	1.27 (0.62–2.61)	3.89 (1.73–8.78)	3.12 (1.59–6.13)	4.70 (1.96–11.26)
Overweight	2.54 (1.10–5.90)	3.86 (1.53–9.73)	5.44 (1.84–16.06)	4.70 (2.02–10.94)

LDL-cholesterol ≥3.0 mmol/L (M: 17.7%; F: 36.3%)				
Low BMI	1	1	1	1
Elevated BMI	2.11 (0.81–5.56)	1.73 (0.93–3.24)	4.17 (1.60–10.88)	1.08 (0.52–2.22)
Overweight	4.00 (1.53–10.42)	1.42 (0.66–3.04)	6.86 (2.15–21.91)	2.52 (1.30–4.86)

HDL-cholesterol <1.03 mmol/L (M: 15.4%; F: 20.0%)				
Low BMI	1	1	1	1
Elevated BMI	0.38 (0.14–1.03)	0.98 (0.48–2.04)	0.33 (0.13–0.84)	0.68 (0.30–1.55)
Overweight	0.27 (0.10–0.75)	2.07 (0.72–5.93)	0.33 (0.10–1.12)	0.76 (0.34–1.68)

Triglycerides ≥1.00 mmol/L (M: 10.9%; F: 13.0%)				
Low BMI	1	1	1	1
Elevated BMI	0.67 (0.17–2.56)	0.86 (0.31–2.38)	2.19 (0.66–7.24)	1.39 (0.37–5.16)
Overweight	1.98 (0.66–5.94)	3.05 (1.18–7.89)	8.03 (2.25–28.64)	7.00 (2.60–18.88)

Glycemia ≥5.6 mmol/L (M: 24.0%; F: 8.8%)				
Low BMI	1	1	1	1
Elevated BMI	0.64 (0.26–1.56)	1.27 (0.39–4.10)	0.86 (0.40–1.84)	1.02 (0.24–4.26)
Overweight	0.90 (0.36–2.24)	3.00 (0.94–9.55)	0.87 (0.29–2.62)	3.27 (1.12–9.50)

Uric acid >0.39 (M) or 0.31 (F) mmol/L (M: 18.9%; F: 20.9%)				
Low BMI	1	1	1	1
Elevated BMI	1.70 (0.69–4.16)	0.90 (0.42–1.94)	1.06 (0.44–2.56)	1.18 (0.48–2.89)
Overweight	1.79 (0.68–4.71)	1.62 (0.70–3.74)	3.81 (1.37–10.59)	2.75 (1.29–5.86)

LDL-cholesterol: low-density lipoprotein cholesterol; HDL-cholesterol: high-density lipoprotein. M: males; F: females.

The prevalence of elevated CRFs in males (M) and females (F) is indicated between brackets.

Low levels of BMI refer to BMI values below the age- and sex-specific medians; overweight (which also includes obesity) is defined according to standard BMI cut-off values; and elevated BMI refers to intermediate BMI values.

**Table 4:** Standardized linear regression coefficients for the associations between cardiometabolic risk factors at the age of 19-20 years and body mass index measured at the age of 12–15 years or at the age of 19-20 years.

	SBP	DBP	LDL-cholesterol	HDL-cholesterol	Triglyceride	Fasting glucose	Uric acid
*Males *							
BMI at 12–15 years	0.24**	0.27***	0.20**	−0.12	0.17*	−0.01	0.27***
BMI at 19 years	0.27***	0.34***	0.26***	−0.16*	0.38***	0.05	0.33***
BMI_12–15_ adj for BMI_19_	0.11	0.07	0.03	−0.03	−0.18	−0.09	0.08
BMI_19_ adj for BMI_12–15_	0.19*	0.28**	0.24*	−0.14	0.51***	0.11	0.28**
*Females *							
BMI at 14 years	0.23**	0.31***	0.04	−0.05	0.14*	0.29***	0.17*
BMI at 19 years	0.31***	0.36***	0.09	−0.17*	0.26***	0.39***	0.27***
BMI_12–15_ adj for BMI_19_	−0.1	0.01	−0.15	0.35**	−0.29*	−0.15	−0.22
BMI_19_ adj for BMI_12–15_	0.39**	0.35**	0.22	−0.47***	0.51***	0.52***	0.46***

adj: adjusted.

*P < 0.05; **P < 0.01; ***P < 0.001.

BMI: body mass index; SBP: systolic blood pressure; DBP: diastolic blood pressure; LDL-cholesterol: low-density lipoprotein cholesterol; HDL-cholesterol: high-density lipoprotein.
